# Staging of lung cancer CT and PET

**DOI:** 10.1186/1470-7330-14-S1-O6

**Published:** 2014-10-09

**Authors:** Theresa C McLoud

**Affiliations:** 1 Department of Radiology, Massachusetts General Hospital/Harvard Medical School, Boston, MA 02114, USA

## 

Lung cancer is the leading cause of cancer related deaths in the United States with a 5 year survival of only 15%. [1] The International Association for the Study of Lung Cancer (IASLC) issued a 7^th^ edition of the TNM staging system for lung cancer in 2007. [2] It includes several revisions which better align TNM staging with prognosis and in some cases with treatment.

There have been revisions in the TNM descriptors. In the T or tumor category, the T1 and T2 categories include now subcategorization of size with new T1a, T1b, T2a and T2b subdescriptors. One of the important changes is that tumors larger than 7 cm are now considered Stage T3 tumors. Stage IV tumors include separate tumor nodules in the same lung but not in the same lobe as the primary lesion which were previously considered metastatic (M1). Stage T4 disease is now downgraded to Stage III when satellite nodules are present in the same lobe as the primary lesion. The presence of malignant pleural effusion, pleural dissemination or pericardial disease is now considered metastatic disease, specifically stage M1a for local intrathoracic disease rather than Stage IV disease [3-5].

Although the IASLC has proposed a new lymph node map there are no changes to the end descriptors in the 7^th^ edition of the TNM staging system [3-5].

Nearly one half of newly diagnosed lung cancers already demonstrate metastases within the lung, brain, liver, and bony structures. Any metastatic disease is automatically designated Stage IV disease and with few exceptions is considered surgically unresectable. The M category is now subcategorized into intra-thoracic metastasis M1a and extra-thoracic metastatic M1b with the former having a better prognosis [4].

Contrast enhanced CT remains the mainstay for staging of lung cancer. However, PET has particular value in nodal staging of lung cancer and also in determining the presence of distant metastatic disease. In a study by Gould et al, the sensitivity of PET CT for metastasis was 85% and the specificity was 95% as compared with a CT sensitivity of 61% and specificity of 79% [6]. PET CT does have a high false positive rate so it cannot replace invasive sampling, but it may be used to direct invasive staging. PET scanning is particularly useful in M staging of non-small cell lung cancer. PET can replace the use of bone scintigraphy and it is now widely used for determination of distant metastasis throughout the body. However, it is limited in the assessment of brain metastases. In the PLUS Trial, 188 patients with potentially resectable non-small cell lung cancer were randomized to either conventional work up or PET CT. Addition of the PET CT to the conventional work up prevented future surgery in 1 out of 5 patients [7].

## References

[B1] American Cancer Society Cancer facts and figures 2008 2008 Atlanta, GA: American Cancer Society

[B2] Goldstraw P Crowley J Chansky K The IASLC Lung Cancer Staging Project: Proposals for the revision of the TNM stage groupings in the forthcoming (seventh) edition of the TNM classification of malignant tumours J Thorac Oncol 2007 2 8 706 714 10.1097/JTO.0b013e31812f3c1a 17762336

[B3] Rami-Porta R Ball D Crowley J The IASLC Lung Cancer Staging Project: Proposals for the revision of the T descriptors in the forthcoming (seventh) edition of TNM classification for lung cancer J Thorac Oncol 2007 2 7 593 602 10.1097/JTO.0b013e31807a2f81 17607114

[B4] Postmus PE Brambilla E Chansky K The IASLC Lung Cancer Staging Project: Proposals for revision of the M descriptors in the forthcoming (seventh) edition of the TNM classification of lung cancer J Thorac Oncol 2007 2 8 686 693 10.1097/JTO.0b013e31811f4703 17762334

[B5] Rusch VW Asamura H Wantanabe H The IASLC lung cancer staging project: A proposal for a new international lymph node map in the forthcoming seventh edition of the TNM classification for lung cancer J Thorac Oncol 2009 4 5 568 577 10.1097/JTO.0b013e3181a0d82e 19357537

[B6] Gould MK Kuschner WG Rydzak BA Test performance of positron emission tomography and computed tomography for mediastinal staging in patients with non-small-cell lung cancer: A meta-analysis Ann Intern Med 2003 139 11 879 892 10.7326/0003-4819-139-11-200311180-00013 14644890

[B7] Van Tinteren H Hoekstra OS Smit EF Effectiveness of positron emission tomography in the preoperative assessment of patients with suspected non-small-cell lung cancer: The PLUS multicentre randomised trial The Lancet 2002 359 9325 1388 1392 11978336 10.1016/s0140-6736(02)08352-6

